# Prevalence of Antiretroviral Therapy Use among HIV/AIDS Patients in a District Hospital

**DOI:** 10.31729/jnma.4618

**Published:** 2019-10-31

**Authors:** Sandeep Gupta, Chandan Kumar Gupta, Shyam BK, Nupur Gupta

**Affiliations:** 1 Department of General Practice and Emergency Medicine, Gandaki Medical College and Teaching Hospital, Pokhara, Nepal; 2 Murarilal Gupta Memorial Hospital, Salampur, UP, India; 3 Department of Internal Medicine, Nepalgunj Medical College and Teaching Hospital, Nepalgunj, Nepal; 4 School of Development and Social Engineering, Pokhara University, Pokhara University, Kaski, Nepal.

**Keywords:** *antiretroviral therapy*, *human immunodeficiency virus*, *opportunistic infections*

## Abstract

**Introduction:**

Prevention of Human Immunodeficiency Virus infection is a high priority for the government of Nepal, so the government has been scaling up Anti Retroviral Therapy centers throughout the country. The objective of our study was to find out the prevalence of people living with Human Immunodeficiency Virus infection on Anti Retroviral Therapy service.

**Methods:**

This descriptive cross-sectional study was done in Lamjung district hospital, from May 2017 till August 2017 after taking ethical clearance from the institutional review committee. The study was done in 96 patients and convenience sampling was done. The data collected was entered in Microsoft Excel and analyzed in Statistical Packages for Social Sciences version 17.0, point estimate at 95% Confidence Interval was calculated along with frequency and proportion for binary data.

**Results:**

Out of the total 109 patients enrolled at the hospital, 85 (78%) were on ongoing Anti Retroviral Therapy. The predominant age group among patients using Anti Retroviral Therapy was 25-34 years 27 (31.7%) and the five most common clinical manifestation/opportunistic infections were fever 40 (47.1%), diarrhea 34 (40%), fatigue/generalized weakness 32 (37.6%), loss of appetite 25 (29.4%) and headache 18 (21.2%) among them. Out of total patients, 14 (12.8%) of our patients were under 14 years of age. We found 71 (83.6%) of the patients continued the original first-line regimen and in 14 (16.5%) one or two drugs were substituted in the original regimen.

**Conclusions:**

Our study showed a similar prevalence of people living with Human Immunodeficiency Virus infection on Anti Retroviral Therapy service with the other studies done within Nepal.

## INTRODUCTION

Human Immunodeficiency Virus (HIV) continues to be a major global public health issue, approximately 37 million people globally living with HIV, over 1.3 million people have died and only 21.7 million accessing Anti Retroviral Therapy (ART) in 2017.^1^

In Nepal, ART service started in 2004 under National Center for Acquired Immune Deficiency Syndrome (AIDS) and STI Control (NCASC) guidelines and presently there are 74 centers throughout the country.^2^ ART has improved the health and has transformed the fatal disease into a chronic condition.^3^ ART service has been started for seven years at Lamjung district hospital and there have been limited studies on ART services in Nepal.

The objective of our study was to find out the prevalence of people living with Human Immunodeficiency Virus infection on ART service.

## METHODS

This descriptive cross-sectional study was carried out at the ART center of Lamjung district hospital, Lamjung, Nepal from May until August 2017 after taking ethical approval from the Institutional Review Committee of Gandaki Medical College for the study. After taking informed consent, patients were interviewed to fill up the pre-structured questionnaire and the records were kept confidential and participation in the research was completely voluntary. The sample size was calculated using the following formula.

$$\begin{array}{l} \text{n}=\frac{{\text{Z}}^{\text{2}}\times \left(\text{p}\times \text{q}\right)}{{\text{e}}^{\text{2}}}\\ ={\left(1.96\right)}^{2}\times \frac{\left(0.5\times 0.5\right)}{{\left(0.1\right)}^{2}}\\ =96 \end{array}$$

where,

n= required sample size
Z= 1.96 at 95% confidence interval
p= prevalence of people living with HIV infection on ART Service (50%)
q= prevalence of people living with HIV infection no on ART Service (1-p)
e= margin of error (10%)

The calculated sample size was 96 and convenience sampling was done. The data collected was entered in Microsoft Excel and analyzed in SPSS (Statistical Packages for Social Services) version 17.0.

## RESULTS

Out of the total 109 patients enrolled at the hospital, 85 (78%) were ongoing ART among which the female to male ratio was 1.2:1. Out of total patients, 14 (12.8%) of our patients were under 14 years of age and 24 (22%) of the patient had been deferred from the therapy including 9 (8.2%) deaths (Table 1).

**Table 1:** Patient enrolled on ART service.

On Anti Retroviral Therapy	n (%)
Adult	
Female	38 (34.9)
Male	33 (30.3)
Children	
Female	9 (8.2)
Male	5 (4.6)
Deferred from ART Services	
Transferred Out	10(9.2)
Lost Follow Up	5 (4.6)
Death	9 (8.2)

**Figure 1:**
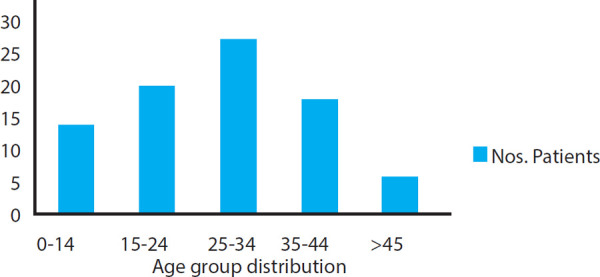
Distribution of patients according to age group.

The predominant age group was 25-34 year which included 27 (31.7%) of patients followed by 15-24 year which included 20 (23.5%) of patients (Figure 1).

The most common clinical manifestation/opportunistic infections were fever 40 (47.1%), diarrhea 34 (40%), fatigue/generalized weakness 32 (37.6%), loss of appetite 25 (29.4%) and headache 18 (21.2%) among our patients (Table 2).

**Table 2:** Clinical presentation/Opportunistic infection of the patients using ART.

Clinical Presentation	n (%)
Fever	40 (47.1)
Diarrhea	34 (40.0)
Fatigue/Weakness	32 (37.6)
Loss of Appetite	25 (29.4)
Headache	18 (21.2)
Pneumonia	14 (16.5)
Weight Loss	14 (16.5)
Gastritis/Reflux	12 (14.1)
Mental Issues	10 (11.8)
Skin Lesion	10(11.8)
Sleeplessness	8 (9.4)
Pulmonary Tuberculosis	6(7.1)

**Figure 2:**
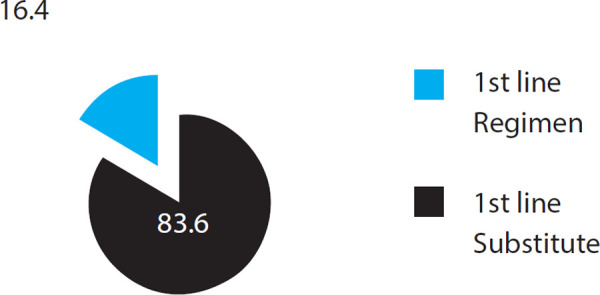
ART regimen continuation.

In the study 71 (83.6%) of patients continued the original first line regimen and in 14 (16.4%) one or two drugs were substituted in the original regimen (Figure 2).

The study showed ART adherence of above 95% on the adherence scale in our patient.

## DISCUSSION

This cross-sectional study was conducted at the ART center of Lamjung District Hospital, Lamjung Nepal and out of the total 109 patients enrolled at the hospital, 78% were on ongoing ART. The National HIV/AIDS strategy 2002-2006 clearly states five priority areas for the prevention of STI and HIV infection within the country. Effective surveillance and efficient management of prevention of HIV/AIDS remains an important element for control of the infection and decrease the morbidity and mortality from AIDS-related illness.^4^ The sixty-ninth World Health Organization (WHO) assembly endorsed a new global health sector strategy on HIV for 2016-2021. The strategy guides priorities action for every country for high priority for the prevention of HIV/AIDS-related infection.^1,5^ At Lamjung district hospital, ART service has been started since 2010, a total of 109 patients had been enrolled for the therapy. Our patients belonged from three different districts-Lamjung, Tanahun and Gorkha as the district hospital is easily accessible to the individuals of these districts. Presently, 85 patients were continuing ART medicines from the hospital. A number of patients undertaking ART at other district hospitals-like Damauli, Syangja, Gorkha were in the range of 65 to 100 as per communication with focal persons of these centers. Our study showed an increasing number of female patients compared to the male patient which differs from the national HIV/AIDS cumulative reported data. There are increasing numbers of male population migrating abroad for working including India, leading to increased infection among females. These findings were similar in other Western and Far Western regions; a study of Dhangadi showed increasing number of female patients compared to males.^6^ The present study at our district hospital revealed that the most common clinical presentations/Opportunistic Infections (OI) were fever (47.1%), diarrhea (40%), fatigue/weakness (37.6%), loss of appetite (29.4%), headache (21.2%) and pneumonia (16.5%). These findings were more suggestive of symptomatic primary HIV infection also known as Acute Retroviral Syndrome. A study from central Nepal showed predominant OIs among PLHA were oral candidiasis (32.0%), streptococcal pneumonia (28.7%), salmonella infection (20.7%), cryptosporidial infection (19.3%), and tuberculosis (10.0%).^7^ In the eastern region of Nepal, study done by Khanal VK et al. showed common OIs were weight loss (74.4%), fever (59.4%), cough (36.3%) and diarrhea (32.5%).^8^ Poudel BN et al. at Seti Zonal Hospital revealed fever (71.7%), diarrhea (56.6%), pneumonia (52.8%), weight loss (52.8%) and oral thrush (33.9%) to be the major clinical presentation/Opportunistic infections.^9^ In studies from India done by Singh S et al. the common clinical features were fever (36.6%), respiratory infections (31.7%), lymphadenopathy (30%), hepatosplenomegaly (21.8%) and diarrhea illness (18%).^10^ The clinical features of admitted HIV infected patients from Pakistan done by Siddique MH et al. at tertiary level teaching hospital showed that the three most common clinical features were weight loss (59.6%), fever (42.3%) and diarrhea (38.8%).^11^ These clinical features/OIs were similar to the presentation of our patients suggesting a similar strain of virus circulating in the region. Effective use of ART is the most useful intervention for suppressing HIV replication, preventing OIs and prolong the survival of PLHA.^12,13^ Our study showed above 95% treatment adherence scale among the PLHA and the findings were similar from a study done at ART center of the western region.^14^

Since this study was done in a small sample size and convenient sampling was done, the findings of this study can’t be generalized. Hence, further study with random sampling and larger sample size is recommended. But the finding may have implications in scaling up ART centers and early initiation of ART at the primary care level for HIV prevention to the eligible patients would be a comprehensive strategy to prevent HIV epidemic progression in developing countries like Nepal.

## CONCLUSIONS

Our study showed a similar prevalence of clinical symptoms and opportunistic infections like fever and diarrhea with the other studies done within Nepal.
